# Guest attendance data from 34 Swedish pre-schools and primary schools

**DOI:** 10.1016/j.dib.2021.107138

**Published:** 2021-05-13

**Authors:** Mattias Eriksson, Christopher Malefors, Luca Secondi, Stefano Marchetti

**Affiliations:** a Department of Energy and Technology, Swedish University of Agricultural Science, Box 7032, 75007, Uppsala, Sweden; b Department for Innovation in Biological, Agro-Food and Forest Systems (DIBAF), University of Tuscia, Via S. Camillo De Lellis, snc, Viterbo (Vt), 01100, Italy; c Department of Economics and Management, University of Pisa, Via Ridolfi 10, Pisa 56124, Italy

**Keywords:** Forecasting, Public sector catering, Food waste, Meal planning

## Abstract

This data article describes 34 datasets, compiled into one table, describing guest attendance at lunch meal servings in Swedish public schools and preschools. Fifteen of the schools and all 16 of the preschools covered belong to one municipality, while the remaining three schools belong to two other municipalities, all located in central Sweden. Data on number of plates was used as a proxy of the number of guests eating lunch. Number of used plates was recorded from late August 2010 to early June 2020, i.e. covering the period both before and during the initial phase of the Covid-19 pandemic, so that making possible to evaluate changes in guest attendance during the pandemic. Since these were real data, all data elements pertaining to exact canteens or staff identity have been removed. There is a scarcity of real business data for scientific and educational purposes, so these datasets can play an important role in research and education within catering management, consumption pattern analysis, machine learning, data mining and other fields.

## Specifications Table

**Table:** 

Subject	Tourism, Leisure and Hospitality Management
Specific subject area	Guest attendance in Swedish public catering.
Type of data	Table.
How data were acquired	Extracted from public Excel files used to record guest attendance by Swedish schools and preschools.
Data format	Mixed (raw and compiled).
Parameters for data collection	Reported zero values were treated as missing data.
Description of data collection	Collected daily in a procedure where catering staff counted and recorded the number of plates used during lunch serving.
Data source location	All schools and preschools are located in the municipalities of Sala, Tierp and Härnösand in central Sweden.
Data accessibility	Data is available at http://dx.doi.org/10.17632/4zr5dwnbbv.1
Related research article	Malefors, C., Secondi, L., Marchetti, S. & Eriksson, M. (2021). Food waste reduction in times of crisis - The potential to forecast guest attendance in Swedish public catering during the covid-19 pandemic, Socio-Economic Planning Sciences. In press. https://doi.org/10.1016/j.seps.2021.101041

## Value of the Data

• The data make it possible to analyse trends and variations within Swedish public sector catering. These data are not normally accessible, since there is no point of sale where data can be easily recorded.
• The data can be used to perform research on different problems, such as forecasting of guest attendance during a pandemic or other types of seasonal sickness (influenza), operations management, seasonality and consumption patterns.
• The data can be used as a basis for forecasting algorithms to avoid overproduction in public catering. Since the data partly cover the Covid-19 pandemic, they can also be used to assess pupil absence during the initial phase of a pandemic as well as to investigate the effects of governmental restraining orders issued.
• Attendance per specific week day to analyse when there is a surge in demand and also to capture absence trends and sickness levels among pupils.
• Analyse differences in attendance for rural and urban schools.
• To be used in nutritional studies so examine how many that take part of the meals and how many who eat elsewhere, which is potentially more “unhealthy”.
• Educators can use the datasets for machine learning classification or segmentation problems
• Educators can use the datasets to obtain statistics or for data mining training.

## 1 Data Description

Swedish public catering provides meals free of charge for pupils in preschools and schools. Since there are no point-of-sales data, records are based on other indicators, such as the number of plates used. However, daily recording of used plates is time-consuming, non-standardised and lacks a supporting system to store the data, which makes this type of information difficult to access even though the records are open to the public. Compilation of the data in a table therefore provides unique insights into public catering guest attendance.

The datasets now made available were collected for the purpose of developing guest attendance forecasting models and specifically to predict guest attendance during a pandemic [1]. However, due to the characteristics of the variables and the long time series included in these datasets, their use goes beyond this initial problem area. The statistical elementary units of analysis are the school distinguished in primary school and preschools units.

Each dataset contains one file with raw data on attendance [2] in compiled form, where the identification code for each school/preschool unit is displayed together with the daily recorded number of used plates and the date of the observation. The attendance data are split into pupils, staff and other guests, which are added together to give total attendance for each day. Each observation is also supplemented with information on total number of students enroled at the school/preschools and comments by the catering staff.

The second file [2] details the dates of holiday periods (when the schools/preschools are open to provide childcare for some pupils), planning days (where only teachers are present in the schools/preschools), weekends and bank holidays (where the schools/preschools are completely closed).

A third file [2] contains information regarding each canteen and specifically:

• The students’ age groups that usually eat there,
• Whether the canteen is a production or satellite kitchen,
• Number of pupils enroled during the school year 2019/2020, when applicable.

Moreover, this file also contains information about contextual characteristics and specifically:

• How far the canteen is from a city centre (calculated according to the Manhattan distance) and
• Whether the canteen is located in an urban or rural setting. This can be used as explanatory variables to detect if there is an option for students to eat elsewhere, which is useful in nutritional studies.

Swedish students have one meal per day for free, but they are not forced to eat within the school canteen and can choose to go and eat elsewhere. However, this might not be practical for students in a rural area simply because there is no alternative place to eat, whereas students who are located in urban areas close to city centre might have more options to choose from and therefore the explanatory variables “distance to city centre” and “rural/urban” might explain some of the absence/presence in attendance. The entire dataset can be used as a benchmark example for implementing attendance analysis in other public or private catering contexts as well as a starting point to further expand the data already collected and the related analysis, by constructing new variables related to the school performance such as staff/pupil ratio as well as to the trend of attendance per weekday, such as the coverage rate of meals served in each school calculated as the number of meals served and the potential number of maximum meals served, a ratio that could be distinguished for students, teachers and school staff.

## 2 Experimental Design, Materials and Methods

The combined dataset table contains guest attendance data as recorded plates for 18 primary school canteens and 16 preschool canteens in Sweden. All preschools and 15 of the schools are located in the municipality of Sala, while the remaining three schools are located in the municipalities of Tierp and Härnösand. The main selection parameter was willingness by the schools/preschools and municipalities to share their records, and thus no random selection of kitchens was performed. All the selected canteens serve meals to pupils ranging in age from around one to 15 years. The material focuses solely on guest attendance during lunch, as this is the most commonly served meal in the selected canteens, although breakfast and snacks may also be served. Some of the school canteens are also responsible for preschools, whose children eat within the establishment, or provide cooked food for other schools. All guests served by a specific canteen were therefore included in the dataset, even though they did not fit into the actual age group of the studied schools/preschools.

Data on number of guests eating school meals were collected from August 2010 to early June 2020, by catering staff in a normal daily routine that involved counting the number of used plates after every lunch in the canteens. The counting procedure is further explained in [4], which also points out that daily plate data for lunch are an approximation of number of guests served, since no point-of-sale data are available as the meals are free of charge. The plate data forms a time series of the guest attendance per school unit. However, guest attendance data are dependant on the school year in Sweden, which consists of at least 178 days between late August and early June [3]. There are four long periods of holiday during the school year. The first holiday week in the autumn term normally takes place at the end of October and the autumn term ends with a winter holiday that lasts approximately three weeks and covers Christmas and the New Year. The spring term has two holiday periods of one week or more, the first in late February and the second around Easter. The summer term contains scattered public holidays in May and early June. Schools normally end their year in mid-July. However, while schools do not provide education during the holidays, they often remain open to provide childcare for younger pupils and therefore serve meals during this period, but with less activity. Preschools are normally kept open during holidays, but guest attendance can still be influenced if families with older siblings use the school holidays for vacation. During the weekend the canteens are closed and thus the attendance has been set to zero.

Information on the canteens were obtained from the public catering manager in each organisation.

## Ethics Statement

The aggregated plate data per day are not linked to individual students or staff.

## CRediT Author Statement

**Mattias Eriksson:** Conceptualization, Methodology, Funding acquisition, Writing – original draft; **Christopher Malefors:** Conceptualization, Methodology, Software, Data curation, Writing – original draft; **Luca Secondi:** Validation, Writing – review & editing; **Stefano Marchetti:** Validation, Writing – review & editing.

## Declaration of Competing Interest

The authors declare that they have no known competing financial interests or personal relationships which have, or could be perceived to have, influenced the work reported in this article.
